# Characterization of *TRZ1*, a yeast homolog of the human candidate prostate cancer susceptibility gene ELAC2 encoding tRNase Z

**DOI:** 10.1186/1471-2199-6-12

**Published:** 2005-05-13

**Authors:** Yang Chen, Audrey Beck, Christina Davenport, Yuan Chen, Donna Shattuck, Sean V Tavtigian

**Affiliations:** 1 Myriad Genetics, Inc. 320 Wakara Way, Salt Lake City, UT 84108, USA; 2 International Agency for Research on Cancer, World Health Organization, 150 Cours Albert-Thomas, 69372 Lyon Cedex 08, France

## Abstract

**Background:**

In humans, mutation of ELAC2 is associated with an increased risk of prostate cancer. ELAC2 has been shown to have tRNase Z activity and is associated with the γ-tubulin complex.

**Results:**

In this work, we show that the yeast homolog of ELAC2, encoded by *TRZ1 *(tRNase Z 1), is involved genetically in RNA processing. The temperature sensitivity of a *trz1 *mutant can be rescued by multiple copies of *REX2*, which encodes a protein with RNA 3' processing activity, suggesting a role of Trz1p in RNA processing *in vivo*. Trz1p has two putative nucleotide triphosphate-binding motifs (P-loop) and a conserved histidine motif. The histidine motif and the putative nucleotide binding motif at the C-domain are important for Trz1p function because mutant proteins bearing changes to the critical residues in these motifs are unable to rescue deletion of *TRZ1*. The growth defect exhibited by *trz1 *yeast is not complemented by the heterologous ELAC2, suggesting that Trz1p may have additional functions in yeast.

**Conclusion:**

Our results provide genetic evidence that prostate cancer susceptibility gene ELAC2 may be involved in RNA processing, especially rRNA processing and mitochondrial function.

## Background

With a cumulative incidence by age 75 of 14% among Caucasian-Americans and 25% among African-Americans, prostate cancer has become the most common non-cutaneous malignancy in the US [1]. Despite its high incidence rate, there is little known about the genetics and etiology of prostate cancer. We recently identified a candidate prostate cancer susceptibility gene through linkage analysis and positional cloning [2]. This gene, ELAC2, is located on chromosome 17p and encodes a protein of 826 amino acids with tRNase Z activity (tRNA 3' endoribonuclease activity) [3]. ELAC2 belongs to an incompletely characterized family of proteins that is conserved among eukaryotes, archaea and bacteria. ELAC2 is ubiquitously expressed in all tissues with the highest expression levels found in testes. Elac2p is detected in several cancer cell lines and binds the γ-tubulin complex [4]. At present the role that sequence variants in ELAC2 may play in the genesis of prostate cancer is unclear.

*TRZ1 *(tRNase Z 1, YKR079c), the yeast ortholog of ELAC2, encodes an essential protein of 838 amino acids. Like ELAC2 and its other eukaryotic orthologs, TRZ1 can be divided roughly into amino- and carboxy-halves, or domains, which are structurally homologous [2]. The carboxy domain possesses tRNase Z activity; the amino domain is enzymatically inactive but may modulate the specificity of carboxy domain. The most strongly conserved sequence element is the histidine motif, located near the front of the carboxy domain. In the amino domain, the structural homolog of the histidine motif is easily recognizable; however, most of the key zinc coordinating histidines have changed to some other polar amino acid, which is entirely consistent with the observation that this domain lacks tRNase Z activity. PROSITE scans revealed that the vertebrate ELAC2s contain a sequence element near the center of their amino domain that matches the nucleotide binding P-loop motif [2]. Although not exactly matching the canonical P-loop sequence, this sequence element is well conserved in TRZ1 and its structural homolog is well conserved near the center of the carboxy domain of all gene family members. It has recently been shown that both of the two ELAC family members (tRNase ZL and tRNase ZS) from the Animals, Plants, Fungi, Bacteria, and Archaea possess tRNase Z activity [3,5-9]. The amino acid and enzymatic conservation across ELAC family members suggests strongly that they all share some basic cellular functions. Since *Saccharomyces cerevisiae *is a well-characterized genetic system, we initiated a genetic analysis of the functions of the yeast member of this gene family, *TRZ1*, in order to better understand those of ELAC2.

RNA processing, one of the post-transcriptional controls on gene expression, plays an important role in regulating cell growth and fate. Pre-mRNA processing is a multi-step process coupled to transcription through the involvement of the unique C-terminal domain (CTD) of the large subunit of RNA polymerase II. The processing involves RNA binding proteins, snRNAs (small nuclear RNAs), endo- and exo-nucleases, and other specific factors. The function of these proteins is subject to regulation by different signaling pathways, according to the developmental stage of the cell [10].

Transfer RNAs (tRNAs) undergo extensive post-transcriptional modifications, including: 5' processing; 3' processing; nucleotide modification; and, in the case of about 20% of yeast tRNA, intron removal in the nucleus before export to the cytosol. RNase P (ribonuclease P), which consists of a RNA subunit and nine proteins, is responsible for 5' tRNA processing (reviewed in [11]). Two proteins, Trz1p [3] and Lhp1p [12], have been shown to be involved in 3' tRNA maturation in yeast; Trz1p has tRNase Z activity while Lhp1p stabilizes pre-tRNAs as an RNA chaperone [3,12]. It is known that there are more than 55 proteins involved in tRNA splicing, modification, degradation and export.

Ribosomal RNAs (rRNAs) are transcribed as large precursors by RNA polymerases I and III in nucleoli [13]. These RNAs also undergo processing, including 5' and 3' cleavages, removal of introns, and nucleotide modifications through the action of a series of RNA/protein complexes. The mature rRNAs complex with about 80 proteins to form ribosomes. The RNase MRP (ribonuclease MRP) complex is responsible for the majority of 5' maturation steps and the exosome complex is responsible for 3' rRNA maturation. Many of the modifications and splicing events require small nucleolar RNAs (snoRNAs) and other specific protein factors. Notably, some proteins are shared between the tRNA and rRNA maturation complexes (reviewed in [14]).

In this paper we show that, although Trz1p is required for growth, yeast are largely insensitive to the absolute level of its expression. ELAC2 cannot functionally replace Trz1p, indicating that Trz1p has additional or specific functions. In the carboxy domain of the protein, the conserved histidine motif and the putative P-loop are important for Trz1p biological function because mutations of the conserved residues in these motifs render the protein inactive in yeast. Through a high copy suppressor screen, we found that *TRZ1 *genetically interacts with *REX2*, suggesting a role for *TRZ1 *in RNA processing and mitochondrial maintenance.

## Results

### Effect of altered *TRZ1 *expression on yeast cell growth

We generated three yeast strain types in order to study the consequences of altered *TRZ1 *expression in yeast. In the first (YL09-01 and YL09-02), *TRZ1 *is expressed from a Gal1 promoter on a low-copy plasmid in a wild type *TRZ1 *background. In the second (YL10-04 and YL10-05), the GAL1 promoter is inserted upstream of *TRZ1 *between the translational start site and its endogenous 5'UTR at the chromosomal locus (no deletion of the endogenous locus). In the third strain type (YL10-01, YL10-02 and YL10-03), endogenous *TRZ1 *is deleted and the gene is expressed from a GAL1 promoter on a low-copy plasmid. The latter two strain types were made to investigate the effect of up- and down- regulation of *TRZ1 *expression on growth. The genotypes of all strains used in this study are listed in Table 1. Also listed in Table 1 is the ρ ^+^/ρ ^- ^status of each strain, assayed as the ability of the strain to grow on media containing only non-fermentable carbon sources. The significance of the ρ ^+^/ρ ^- ^status, which represents the wild type (ρ^+^) or impaired (ρ^-^) (petite mutant) mitochondrial respiratory function, will become clear below.

**Figure 1:**
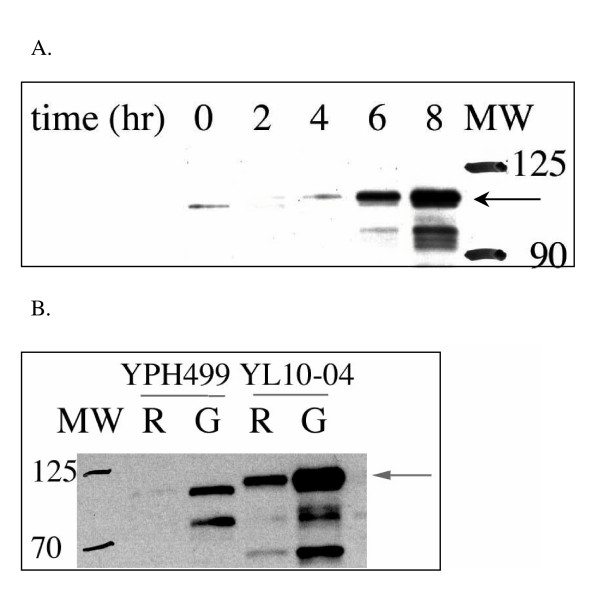
Induction of exogenous FLAG•Trz1p. A. Growth of YL09-02 was initiated in raffinose complete medium. After overnight growth at 30°C, the culture was diluted with raffinose complete medium to O.D. 0.2–2.3 at 600 nm, grown to O.D. 0.4, then induced in exponential growth phase with 2% galactose for varying periods of time before processing for Western blot. B. YPH499 and YL10-04 were grown as described above and induced with 2% galactose for 4 hours before harvesting for Western blot. Arrow indicates FLAG•Trz1p.

As shown in Table 2, when *TRZ1 *is over-expressed in a wild type background (YL09-01), the yeast doubling time is 3.7 hours (average of 2 assays, 3.85 hours in one experiment and 3.55 hours in another experiment), not significantly longer than that of YPH499, the parental yeast control (3.4 hours; average of 2 assays, 3.11 hours in one experiment and 3.75 hours in another experiment) or that of wild type yeast that over-express β-galactosidase (3.0 hours, one assay only). Therefore, over-expression of *TRZ1 *in wild type yeast does not significantly slow yeast growth. Overexpression of FLAG-*TRZ1 *in YL09-02 was verified by Western blot with anti-FLAG antibody. As shown in Figure 1a, FLAG -*TRZ1 *is rapidly induced in YL09-02 grown on galactose-containing medium. Notably, both YL09-01 and YL09-02 are ρ^+^.

We then investigated the consequence of over-expression of *TRZ1 *from the chromosomal locus. In YL10-04 a GAL1 promoter is inserted immediately upstream of the translational start codon of FLAG-*TRZ1*. As shown in Figure 1b, FLAG-*TRZ1 *is promptly induced after addition of galactose in the YL10-04 strain. Liquid growth assays in a variety of different media were done to determine the doubling time of strains expressing varying levels of Trz1p (Table 2). No growth was observed for YL10-04 and YL10-05 in galactose medium, respectively, compared to 3.75 hr doubling time forYPH499. However, these GAL1 promoter insertion strains also grew more slowly in non-inducing medium. The doubling time for YL10-04 and YL10-05 in glucose medium is 3 and 2 times longer than that of parental YPH499 cells (Table 2). We experienced difficulties when trying to isolate Gal1 promoter insertion strains and found that the isolated strains were petite mutants; therefore, we decided not to study these strains further.

We observed the growth characteristics of *trz1 *strains with *TRZ1 *expressed from a GAL1 promoter on a low-copy plasmid (YL10-01, YL10-02 and YL10-03). As shown in Table 2, when *TRZ1 *expression is repressed in glucose medium, YL10-01 grows marginally slower (doubling time = 2.95 hours) than YPH500, the isogenic wild type yeast (doubling time = 2.27 hours). The same is true for YL10-02 and YL10-03 with respect to YPH499, their isogenic wild type strain. Thus, although *TRZ1 *is an essential gene, even low levels of its transcript are sufficient for near normal growth. When *TRZ1 *expression is induced in galactose medium, however, YL10-01 and YL10-02 fail to grow, while YL10-03 grows at about the same rate as wild type yeast. This is intriguing, particularly when comparing YL10-02 and YL10-03, because the strains share the same genetic background. They differ in two ways: YL10-02 is ρ^- ^(petite phenotype) while YL10-03 is ρ^+ ^(table 1); and YL10-02 expresses untagged *TRZ1 *while YL10-03 expresses *TRZ1 *tagged with a FLAG epitope. The phenotypic discrepancy between YL10-02 and YL10-03 is not likely due to a lack of expression of Trz1p or lack of functional Trz1p in YL10-03, because we could detect FLAG•TRZ1p in YL10-03 (data not shown) and p414/Gal1p-FLAG•TRZ1 could functionally complement the *trz1 *deletion (Table 4). The difference between the strains is more likely due to their ρ status.

We studied these strains further by testing the effect of different carbon sources on their growth. Table 3 summarizes these results. When *TRZ1 *expression from a Gal1p-driven *TRZ1 *plasmid is suppressed on SC-glucose, wild type yeast (YL09-01) and *trz1 *yeast (YL10-03) grow at a rate similar to that of YPH499 at room temperature, 30°C and 37°C. We could not detect any significant difference in growth rate among YPH499, YL09-01 and YL10-03 on sucrose, raffinose or galactose at any temperature tested (Table 3). This result agrees with the results from liquid growth assays in that over-expression of *TRZ1 *in these two strains (YL09-01 and YL10-03) does not result in any discernable change in growth rate. When spotted onto glucose media and incubated at room temperature, YL10-01 and YL10-02 grow at a rate equivalent to that of YPH499. As the incubation temperature is increased, however, YL10-01 and YL10-02 grow significantly slower than YPH499. When spotted on sucrose media, these strains grow slower than YPH499 at all tested temperatures, with the most significant difference in doubling time occurring at 37°C. YL10-01 and YL10-02 barely grow on raffinose and do not grow at all on galactose. As argued above, the phenotypic discrepancies among the strains is not likely due to differences in expression of functional TRZ1p. Instead, the different phenotypes resulting from galactose-induced expression of Trz1p is more likely attributable to the ρ^+^/ρ^- ^status of the strains, consistent with the known inability of ρ^- ^cells to grow on galactose-containing media [15]. Thus, the ρ^- ^stains (YL10-01, YL10-02, YL10-04 and YL10-05) show sugar and temperature sensitivity; the ρ^+ ^strains (YL09-01 and YL10-03) do not. In summary, yeast are largely insensitive to the level of Trz1p, provided there is some minimal level of the protein; the apparent sensitivity of some strains to galactose-induced Trz1p expression is attributable to the non-specific galactose-sensitivity of ρ^- ^yeast.

### ELAC2 does not functionally substitute for *TRZ1; *motifs required for Trz1p function

Since *TRZ1 *is the yeast homolog of ELAC2, we tested the ability of ELAC2 to functionally complement deletion of *TRZ1 *in yeast. YL03-47 was transformed with an array of ELAC2 constructs (full length ELAC2, FLAG tagged ELAC2, N-domain ELAC2, C-domain ELAC2, and four full length ELAC2 variants found in prostate patients) (Table 4). Transformants were plated onto FOA to select against the *TRZ1 *expression plasmid and to determine if any construct could complement *trz1*. As shown in Table 4, neither wild type nor prostate cancer-associated ELAC2 mutants could substitute for wild type *TRZ1 *function in YL03-47. The human paralog of ELAC2, ELAC1, likewise failed to rescue *trz1 *(Table 4).

**Figure 2:**
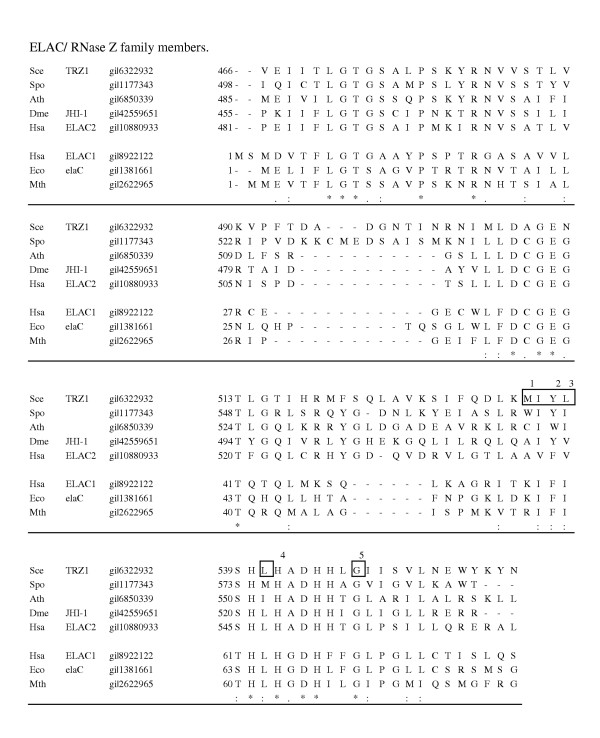
Protein multiple sequence alignment across the core histidine motif of selected ELAC/ tRNase Z family members. The ELAC/tRNase Z family members were selected from *Saccharomyces cerevisiae *(Sce), *Schizosaccharomyces pombe *(Spo), *Arabidopsis thaliana *(Ath), *Drosophila melanogaster *(Dme), *Homo sapiens *(Has), *Escherichia coli *(Eco) and *Methanothermobacter thermautotrophicum *(Mth). The boxed residues in the *S. cerevisiae *sequence are the amino acids that we mutagenized in our effort to create a ts mutant. 1, *S. cerevisiae *M535 corresponds to human A541. 2, Y537L is ts. 3, L538K is ts. 4, H542 corresponds to human H548, the position of a prostate cancer associated frameshift. 5, G548 corresponds to G256 of *S. cerevisiae *PSO2; although PSO2 G256R is a ts mutant [16], TRZ1 G548R is lethal. * denotes identical amino acid, and : /. indicates homologous conservation across species.

We next incorporated three specific mutations into the histidine motif region of *TRZ1*: (i) M535T, which corresponds to the missense substitution A541T in ELAC2 that is associated with human prostate cancer; (ii) H542ter, which corresponds to the frameshift 1641insG associated with human prostate cancer; and (iii) G548R, which corresponds to a ts mutant, G256R, that was isolated in the homologous yeast gene PSO2 [16] (Figure 2) (*trz1 *M535T, *trz1 *H542Ter, *trz1 *G548R; Table 4). Notably, in no case did the fusion of two copies of the FLAG epitope to *TRZ1 *alleles influence results (Table 4). The missense mutant *TRZ1 *allele (*trz1 *M535T) complements *trz1 *as well as the wild type gene. However, neither *trz1 *G548R in which a conserved glycine in the histidine motif is altered, nor the mutant *TRZ1 *truncated after His542 in the same motif (*trz1 *H542Ter) could complement *trz1 *in yeast.

Motif searches identified a putative P-loop (A/GxxxxGKS/T) at amino acids 276–283 of human ELAC2 (AxxxxGKS). This sequence element is located near the center of the N-domain and is clearly conserved among the eukaryotic orthologs. The corresponding sequence in Trz1p (AxxxxGQT, residues 224–231) falls within the range of variation for the structural P-loop recently explored by Brakoulias et al [17]. In the homology between the N- and C-domains of the protein, this candidate P-loop corresponds to a strongly conserved element near the middle of the C-domain (residues 693–700 of Trz1p), which also has features reminiscent of the structural P-loop [17]. For simplicity, we will refer to these as the first and second putative P-loops. In so doing, however, we recognize that they may be examples of some other conserved structural element. We mutated the key residues in both putative P-loops individually and tested the mutants' abilities to complement *trz1*. The first putative P-loop is dispensable for survival because mutants *trz1 *G229D and *trz1 *Q230A, which should destroy its function if it is a P-loop [18,19] (and are far outside the cross-species range of variation of the sequence element if it is not a P-loop), can functionally substitute for the essential function of Trz1p. However, the second putative P-loop is required for yeast survival because mutants here (*trz1 *G698D and *trz1 *D699A) cannot complement *TRZ1 *deletion. Sequence analysis shows that *TRZ1 *may be the result of ancient tandem duplication/fusion that produced a gene with homologous amino and carboxyl halves. We therefore tested whether either the N- or C- domain of *TRZ1 *alone is capable of rescuing *trz1*. Neither half could rescue (FLAG.*TRZ1*.C-term and FLAG.*TRZ1*.N-term), even when fused to the complementary domain of ELAC2 (FLAG.TRZ1-ELAC2 and ELAC2-TRZ1) (Table 4).

### Screen for and characterization of temperature sensitive (ts) *TRZ1 *mutants

**Figure 3:**
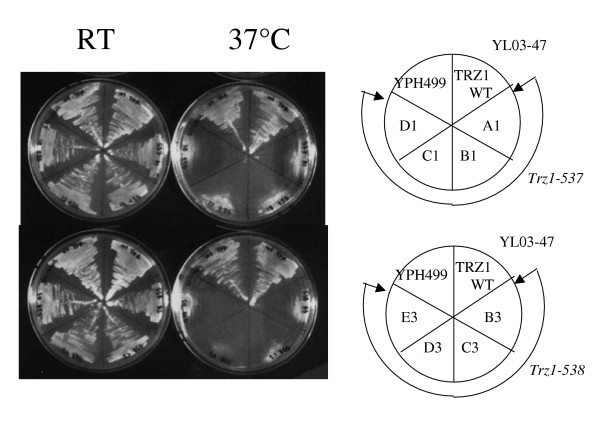
Temperature sensitive growth of yeast expressing alleles *trz1-537 *and *trz1-538*. Isolates of yeast expressing *trz1-537 *(A1, B1, C1, D1) or *trz1-538 *(B3, C3, D3, E3) (YL03-47 with ts alleles on a *LEU2 *plasmid) were streaked onto SC-LEU plates and incubated at RT or 37°C. YPH499 and YL03-47 were used as controls.

We screened for ts mutants of Trz1p with PCR-based random mutagenesis and bacterial mutator approaches; neither of these attempts identified ts mutants. To circumvent technical difficulties, we used a targeted approach instead. We designed degenerate oligos targeting sequence encoding amino acids 535, 536, 537, 538, 541 and 548 in the Trz1p histidine motif (Figure 2). These amino acids were selected for mutagenesis because, with the exception of residue 548, they are less conserved across species than the canonical residues of the histidine motif; therefore, mutation may be less likely to completely inactivate protein function (Fig. 2). G548 was targeted because, as mentioned before, a ts mutant had been identified at the corresponding amino acid in S. cerevisiae PSO2. We generated 6 mini-libraries, each of which targets one of these residues in YL03-47, and isolated the clones that appeared at 30°C but that grew slowly or not at all at 37°C. To confirm the ts phenotype plasmids from these isolates were purified and used to transform naïve YL03-47. The screen identified two ts mutants of Trz1p, Y537L and L538K (Figure 3). We also found mutations at position 535, 536, 537, 538, 541 and 548 that are lethal at both 30°C and 37°C. Observation of lethal missense substitutions at M535 is intrinsically interesting because it is consistent with the idea that the corresponding residue A541 of human ELAC2 is under some functional constraint. More than 60% of the lethal mutations were concentrated at G548, indicating that this residue is more functionally constrained than the corresponding G256 of *S. cerevisiae *PSO2 (data not shown).

**Figure 4:**
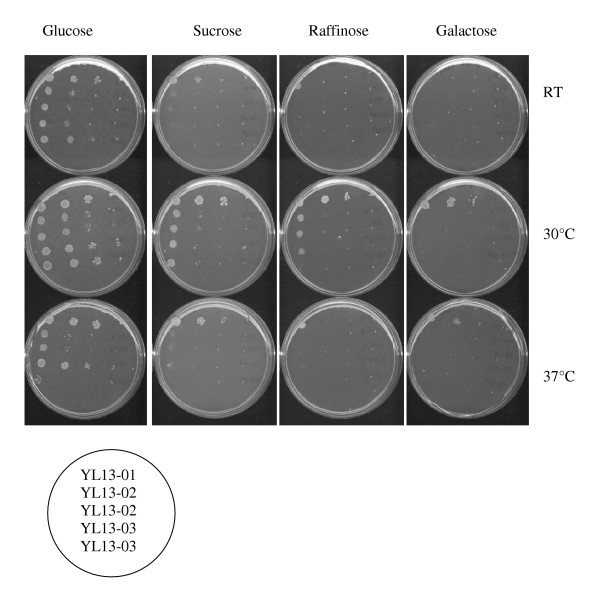
Growth at different temperatures of yeast expressing *TRZ1*, *trz1-537 *or *trz1-538 *alleles on media with various carbon sources added. Yeast bearing *TRZ1 *alleles integrated at the *LEU2 *locus [YL13-01 (WT), YL13-02 (*tr1-537*) and YL13-03 (*trz1-538*)] were serially diluted and spotted onto SC-LEU with different sugars added. Plates were incubated at RT (upper), 30°C (middle) and 37°C (lower).

We integrated *TRZ1*, *trz1-537 *(encodes Trz1p Y537L) and *trz1-538 *(encodes Trz1p L538K) into the *LEU2 *locus in a *trz1 *background to yield YL13-01, YL13-02, and YL13-03, respectively. As shown in Figure 4, the ts mutants grew more slowly on glucose medium than did the controls at all temperatures tested. The inhibition of growth was more obvious at higher temperature. Growth of the ts mutants was further slowed on sucrose. When plated on raffinose, ts mutants grow only at 30°C. YL13-02 and YL13-03 are also petite mutants (i.e., ρ^-^) while YL13-01 is not (Table 1). The ability of YL13-01 but not YL13-02 or YL13-03 to grow on galactose can therefore be explained in terms of the known inability of ρ^- ^yeast to grow on galactose [15]. Table 5 summarizes the doubling time for each ts mutant at different temperatures in SC-Leu medium containing glucose. At 30°C YL13-02 and YL13-03 grow at about the same rate in liquid medium containing glucose as does YL13-01. However, at 37°C the ts strains grow significantly more slowly in glucose (2–3 fold increase in doubling time) than does YL13-01.

**Figure 5:**
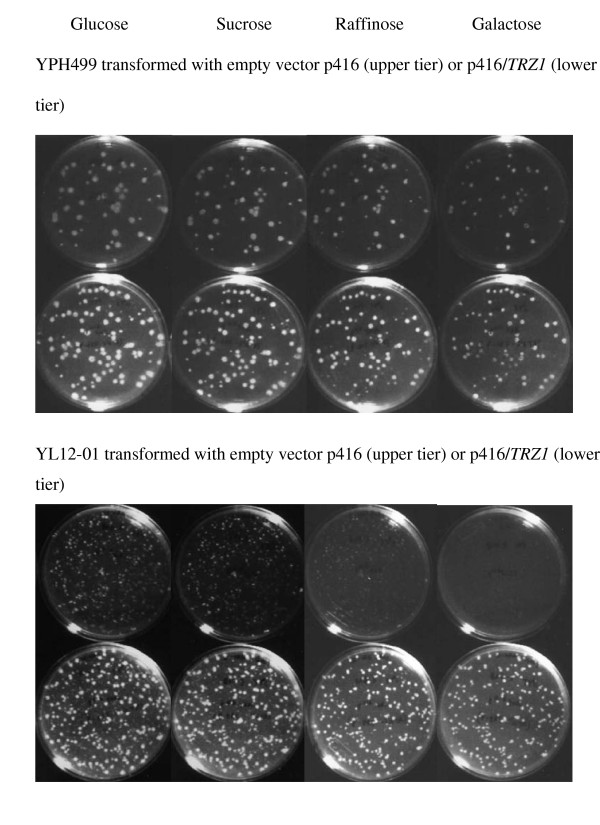
Wild type *TRZ1 *restores growth at 37°C of YL12-01 on media supplemented with different sugars. YPH499 (A) and YL12-01 (B) were transformed with either an empty vector (upper plates) or a vector bearing the wild type *TRZ1 *gene (lower plates). Transformants were replica-plated onto SC-URA containing different sugars and incubated at 37°C for 2 days. Wild type *TRZ1 *restores the growth of ts strain YL12-01 on all carbon sources.

We integrated the *trz1-537 *allele at the *TRZ1 *locus to yield YL12-01. YL12-01 exhibits a slow-growth phenotype like that of the *LEU2 *integrants (YL13-02 and YL13-03). YL12-01 grows slower on glucose media compared with the isogenic wild type YPH499 at all temperatures tested and this growth difference increases at 37°C (data not shown). The *trz1-537 *integrants exhibit severe ts growth on sucrose containing medium and they do not grow on raffinose and galactose containing medium (data not shown). The doubling time of these mutants in glucose-supplemented liquid medium is in agreement with results from the plate assay (Table 5). To verify that the growth phenotype and sugar sensitivity observed with *trz1-537 *integrants do not result from a cloning artifact, we did the following. YPH499 and YL12-01 were transformed with empty vector (p416) or p416/*TRZ1 *and plated onto SC-Ura containing glucose. Once grown, colonies were replica-plated onto SC-Ura supplemented with glucose, sucrose, raffinose or galactose. Providing a wild type *TRZ1 *on a CEN vector restored the growth of YL12-01 to the level of YPH499 on all media tested (Figure 5). YL12-01 transformed with an empty vector grew more slowly than YPH499 on all media tested, with the decrease in growth rate in the order of glucose, sucrose, raffinose and galactose media, in agreement with the previous plate assay. In addition, we checked the ρ status for these transformants. Providing a copy of wild type *TRZ1 *on a CEN vector (YL12-02) converted YL12-01 from ρ^- ^to ρ^+ ^(Table 1), indicating that the petite phenotype of YL12-01 may not be due to the loss of mitochondrial DNA. Instead, it may result from the interference of important mitochondrial functions, suggesting that Trz1p also plays a role inside mitochondria. However, normal growth on a non-fermentable carbon source was not restored in diploid strains derived from crosses between ts mutant strains and a haploid ρ^0 ^tester strain (Table 1 and data not shown). Given the fact that the tester strain should have a wild type copy of *TRZ1 *and ts mutant haploid strains should have mitochondrial DNA, we speculate that ts alleles of *TRZ1 *may have pleiotropic effects on certain important activities in mitochondria. The effect on some of these activities may be dominant, and mating with a wild type haploid strain lacking mitochondrial DNA may not fully restore the mitochondrial functions.

To determine if the *trz1-537 *allele causes any cell cycle arrest phenotype, we analyzed YL12-01, with YPH499 as control, by FACS; both strains were grown at RT or 37°C for this study. Allele *trz1-537 *caused a 1 hour delay in cells entering 2N (M phase) at 37°C. When yeast reached stationary phase, YL12-01 had more cells in 2N (M phase) than YPH499 grown under the same conditions (data not shown). When viewed under a microscope the *trz1-537 *integrants were observed to aggregate when grown at 37°C while parental YPH499 cells did not. With YL12-01 we also observed enlarged cells and cells with elongated buds; the percentage of these abnormally shaped cells increased with the length of time that the cells were grown at 37°C (data not shown). Therefore, *trz1-537 *allele does not cause any cell cycle arrest phenotype and the abnormal shape we observed is most likely the result of cell death in yeast.

### High copy suppressor screen

**Figure 6:**
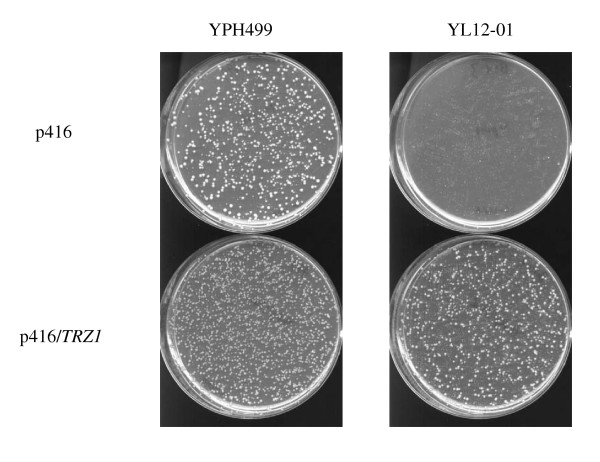
Growth difference exploited for suppressor screen. YPH499 and YL12-01 were transformed with either an empty vector (p416) or with p416/*TRZ1 *expressing a wild type *TRZ1 *gene. Transformants were plated onto SC-URA and incubated at 30°C for 3 days.

During the characterization of YL12-01, we identified a window of two days wherein it would be possible to screen for high copy suppressors of *trz1-537*. As shown in Figure 6, at 30°C, the appearance of YL12-01/p416Gal1 transformants takes 4 days on SC-URA. However, colonies appear after only 2 days when the same strain is transformed with p416/*TRZ*1 (YL12-02). YPH499 transformed with vector or *TRZ1 *resulted in observable colonies after 2 days of incubation at 30°C on SC-URA. Therefore, we screened for high copy suppressors of *trz1-537 *by using yeast genomic fragments to transform YL12-01 and selecting transformants that had grown out in 2-3 days. We screened approximately 34,000 genomic fragments and isolated 4 suppressor clones. Two of the four clones yielded an identical sequence from a 8057 bp genomic region on chromosome 12 (253,621-261,678) that contains four genes (*ERG3*, YLR057w, *SHM2 *and *REX2*). One of the remaining clones includes a 2594-bp genomic sequence from chromosome 11 that contains the full-length coding region of *TRZ1 *plus 25 bp of 5'UTR and 53 bp of 3'UTR. The 4^th ^clone contains chromosome 12 sequences at one end (from 794,633 down) and Ty3 LTR at the other. This last clone was not pursued further.

We cloned each of the four genes on the 8057 bp region of chromosome 12 and inserted these independently into CEN and 2 μ expression vectors containing the CYC1 promoter. We tested each gene for an ability to rescue the ts phenotype of *trz1-537 *yeast. Only *REX2 *rescued the ts phenotype and rescue was observed with both CEN and 2 μ vectors. REX2 encodes a RNA 3' processing enzyme that localizes to mitochondria [20,21].

## Discussion

In this paper, we report investigation of *TRZ1*, the yeast homolog of ELAC2. Altered expression of *TRZ1 *in yeast does not cause any altered growth phenotype. Conservation of the histidine motif and the C-domain putative P-loop is important for the essential function of Trz1p in yeast. Trz1p (Y537L), a ts mutant, exhibits a slow growth phenotype on a variety of yeast media. Additional copies of *REX2 *rescue the slow growth phenotype associated with Trz1p (Y537L), providing genetic confirmation that the product of *TRZ1 *plays a role in RNA processing.

### 1. Altered expression of *TRZ1 *is not detrimental to yeast growth

Although *TRZ1 *is an essential gene, yeast can tolerate altered expression of *TRZ1*. We down-regulated *TRZ1 *message by deleting the endogenous gene and expressing *TRZ1 *from a Gal1 promoter on a CEN plasmid. We could not detect any apparent growth defect when *TRZ1 *expression was suppressed on glucose-containing medium. We also over-expressed exogenous *TRZ1 *message and protein from a Gal1 promoter on backgrounds of either an intact or deleted endogenous *TRZ1 *locus. In neither of these backgrounds does over-expression of *TRZ1 *cause any significant alteration of yeast doubling time. We also attempted to decrease *TRZ1 *mRNA by integration of a thermal labile cassette (Degron approach; data not shown) or by insertion of a Gal1 promoter just upstream of the endogenous *TRZ1 *coding sequence. In these attempts, however, we did not obtain any integrants that were not petite mutants.

In conclusion, under or overexpression of TRZ1 does not grossly affect growth or cell cycle; however, the tendency for alteration in this gene to cause a petite phenotype confounds our ability to detect small perturbations.

### 2. ELAC2 cannot substitute for *TRZ1 *function and the C-domain putative P-loop is important for *TRZ1 *essential function

Human ELAC2 cannot rescue *TRZ1 *deletion in yeast. We observed this with both full-length ELAC2 and with *TRZ1*/ELAC2 and ELAC2/*TRZ1 *fusions. The same lack of complementation was observed with ELAC1, the paralog of ELAC2. These results could be explained if ELAC1 and ELAC2 have overlapping but not totally redundant functions. *TRZ1 *is more similar to ELAC2 than to ELAC1, but Trz1p may accomplish functions in yeast performed by both ELAC1 and ELAC2 in humans. Therefore, substituting *TRZ1 *with either one of these genes may not replace all of the essential functions of Trz1p in yeast. It would be interesting to see whether supplying both human ELAC1 and ELAC2 could rescue *TRZ1 *deletion in yeast.

A motif search found two putative nucleotide-binding motifs (P-loops) in *TRZ1 *and ELAC2, one in the N-domain and one in the C-domain of each gene [2]. The finding that yeast tolerates mutation of the N-domain but not the C-domain P-loop of *TRZ1 *suggests the putative C-domain nucleotide-binding motif plays an important role in Trz1p function. The fact that the C-domains of *TRZ1 *and ELAC2 contain the most conserved sequence across other organisms also supports the notion that the vital functions of *TRZ1 *require that this sequence element is intact. However, the N-domain of Trz1p is also necessary for function because expression of an N-terminally deleted *TRZ1 *does not rescue *trz1 *yeast, nor did the expression of the fusion protein between the N-domain of human ELAC2 and the C-domain of yeast *TRZ1*. This observation is consistent with the results from Nashimoto's lab [9]. Their data suggest that the N-terminal half of ELAC2 (human tRNase ZL), to which family yeast *TRZ1 *also belongs, is responsible for substrate specificity and confers RNase 65 activity that is not present in ELAC1 (human tRNase ZS). And the activities of tRNase Z and RNase 65 toward a set of substrates between human ELAC2 and yeast *TRZ1 *are different, suggesting the N-domain of tRNase ZLs is essential for certain functions in the cell and they may not functionally equivalent among different species [9].

### 3. Potential role of *TRZ1 *in rRNA biogenesis, mRNA splicing and mitochondrion maintenance in addition to its role in tRNA processing

The majority of the RNA in growing yeast is noncoding [13] including rRNA, tRNA, snRNA, snoRNA as well as the RNA components of RNase P and RNase MRP. These RNAs are involved in processes that are vital to cell survival. The biogenesis of rRNA is a complicated process that involves a large number of proteins and RNAs that catalyze or modulate transcription, translocation, and various processing reactions, including exonucleolytic and endonucleolytic cleavages, ligations, terminal additions (5S), and nucleoside modifications. The details of yeast rRNA processing and some of the complexes involved have been described [13,14,22,23].

REX proteins (RNA exonucleases) belong to a family of well-conserved RNA 3' → 5' exonucleases [24]. Rex1p is required for 5S and tRNA-Arg3 maturation. Deletion of Rex2p results in a defect in the processing of the U4 snRNA that is involved in mRNA splicing. Rex3p is required for the maturation of MRP RNA, an important endonucleolytic complex involved in processing both rRNA and tRNA. Rex1p and Rex2p have been shown to be involved in the final maturation of 5.8S rRNA. Rex1p, Rex2p and Rex3p are also involved in the maturation of U5 snRNA and the RNA subunit of RNase P, the RNase that is involved in both tRNA 5' and rRNA maturation. In addition to participating in tRNA and rRNA processing, Rex2p has been implicated in spliceosome assembly by association with the snRNP U2 component PRP11, in DNA repair by association with Rad50p, and in cytoskeleton reorganization by association with the actin cortical patch component Cof1p [25]. Rex2p localizes to the mitochondria and interacts genetically with *YME1 *and *YME2*, components of the inner mitochondrial membrane that participate in mitochondrion organization and biogenesis [20,21]. In summary, the REX proteins function in both RNA maturation and mitochondrial maintenance. The finding that *REX2 *rescues the slow growth phenotype of yeast expressing Trz1p (Y537L), suggests that *TRZ1 *may also participate in those same processes. Further support for this hypothesis comes from the finding that *TRZ1 *exists in a complex with *NUC1 *[26,27], an RNase and DNA endo/exonuclease that localizes to the mitochondria and is involved in mitochondrial DNA recombination. In addition, we observed that supplying an extra copy of wild type *TRZ1 *on a CEN plasmid alters YL12-01, converting the ts mutant Trz1p (Y537L) from ρ^- ^to ρ^+ ^(Table 1). The mitochondrial localization of Trz1p [26] together with the suppression of the Trz1p (Y537L) phenotype by *REX2 *and the effect of expression of wt *TRZ1 *on the petite mutant status of YL12-01 suggest that Trz1p may participate in the activities of mitochondria.

The human homolog of *TRZ1*, ELAC2, was identified as a candidate prostate cancer susceptibility gene [2]. Schiffer et al. [5] purified a nuclear tRNase Z from wheat germ and found two homologs of tRNase Z in *A. thaliana*, *NUZ *(nuclear RNase Z) and *CPZ *(chloroplast RNase Z). Both *Arabidopsis *homologs have tRNase Z activity *in vitro*. These authors also showed that *NUZ *is a homolog of ELAC1 and represents a family of proteins that is conserved from bacteria to humans. Subsequently, Takaku *et al*. showed that human ELAC2, its paralog ELAC1, and the yeast homolog *TRZ1 *have tRNase Z activity *in vitro *[3]. The finding in this study that extra copies of *REX2 *rescue a ts allele of *TRZ1 *confirms a role for Trz1p in the processing of RNA, placing *TRZ1 *in particular in rRNA and tRNA maturation pathways. This is consistent with previous results. First, rRNA biogenesis involves processes both in the nucleolus and cytoplasm, and *TRZ1 *protein localizes to both of these cellular compartments [28]. Second, many proteins and some complexes, for example, MRP, RNase P and the RNA polymerase III complex, participate both in tRNA and rRNA processing and synthesis. Therefore it is possible that *TRZ1 *participates in both tRNA and rRNA processing. Third, using a tet-promoter *TRZ1 *allele and a custom DNA oligo array for noncoding RNA biogenesis, Peng *et al*. demonstrated that *TRZ1 *has an undefined role in the processing of 35S rRNA [29]. The high-copy suppressor result could also be explained more straightforwardly if we assume that Trz1p (Y357L) has diminished or abolished tRNase Z activity, which could result in low level of mature tRNAs, and Rex2p could remove the 3' trailers from pre-tRNAs exonucleolytically, that could result in restored mature tRNA levels.

Since ELAC2 cannot functionally complement *TRZ1 *deletion, we cannot directly test in yeast the functional effect of the human ELAC2 missense substitution A541T, which appears to confer modest risk of prostate cancer [30]. Although this variant does not seem to affect the tRNase Z activity of human ELAC2 in vitro [3], we did find that some missense substitutions at the corresponding yeast residue, M535, are lethal, demonstrating that this position is under functional constraint. Recent demonstrations that *hoe-1*, the *C. elegans *homolog of ELAC2, plays a role in germline proliferation [31], and human ELAC2 maybe involved in cell cycle regulation [4], may help better illuminate the contribution of ELAC2 to the etiology of human prostate cancer.

## Conclusion

We have undertaken a genetic study of yeast *TRZ1 *gene, the homolog of prostate cancer susceptibility gene ELAC2. Our data suggest that the absolute level of *TRZ1 *transcript is not important for the fitness of yeast, the vital functions of *TRZ1 *require an intact C-terminal P-loop and some conserved residues in Histidine motif. More importantly, our high copy suppressor screen suggests that *TRZ1 *is involved in RNA processing, especially rRNA processing and mitochondrial biogenesis.

## Methods

### Strains and Reagents

The strains used in this study are listed in Table 1. The petite status of each strain, determined by growth on a non-fermentable carbon source, is listed in Table 1. YPH499, YPH500, and YPH501 were purchased from Strategene (La Jolla, CA). Genetic manipulations, yeast growth conditions and preparation of yeast media and plates were as described [32]. Yeast transformation and gene disruption were carried out using lithium acetate [32]. Cloning and manipulation of yeast plasmids were done in bacterial strain DH5α. Yeast medium was purchased from Qbiogene (Carlsbad, CA). 5'-Fluoroorotic acid (5'-FOA), used at a concentration of 0.1%, was purchased from Toronto Research Chemicals, Inc. (Ontario, Canada).

### Plasmid and strain construction

A 3.7 kb fragment containing *TRZ1 *coding sequence and intergenic regions was amplified from yeast genomic DNA (primer sequences available upon request) and cloned into pBluescript-KS (BSKS/*TRZ1*). To express epitope-tagged protei*n*, *TRZ1 *N- and C-termini were re-engineered using PCR. We generated BSSK *TRZ1*•FLAG (2 copies of a FLAG epitope fused to the C-terminus), BSSK/*TRZ1*•V5 (1 copy of a V5 epitope fused to the C-terminus), BSSK/*TRZ1*•3XHA or BSSK/*TRZ1*•3Xmyc (3 copies of HA or Myc epitopes fused to the C-terminus, respectively) and BSSK/FLAG•*TRZ1 *(2 copies of a FLAG epitope fused to the N-terminus). Site-directed mutagenesis was performed with QuikChange Site-Directed Mutagenesis kit from Stratagene (La Jolla, CA) following their protocols.

YL03-47 is a *trz1 *haploid strain in which the genomic *trz1 *is complemented by high-copy episomal TRZ1. To construct this strain, first a *TRZ1/trz1 *heterozygote, was generated using a DNA fragment (*TRZ1•hisG•URA3•hisG•TRZ1*) that contained in succession: the *TRZ1 *promoter region; the 5' 37 codons of *TRZ1*; *URA3 *flanked by *hisG *cassettes; the 3' 123 codons of *TRZ1*; and 108 bp of sequence 3' of the *TRZ1 *stop codon. YPH501 was transformed with this fragment, and *TRZ1/trz1 *heterozygotes were selected as uracil prototrophs. Next, one such *TRZ1/trz1 *heterozygote was plated onto FOA to select a *URA3*-deficient derivitive of the *TRZ1/trz1 *heterozygote. This *TRZ1/trz1 *uracil auxotroph was then transformed with a 2 μ *URA3 TRZ1 *expression plasmid (pRS426/*TRZ1*). Finally, to obtain YL03-47, tetrads from the resulting transformants were dissected, and URA+ colonies were screened by PCR for the presence of *hisG *sequence at the endogenous *TRZ1 *locus.

YL12-01, in which the chromosomal *TRZ1 *is replaced with *trz1-537 *allele, is constructed from YPH499 following a two-step gene replacement strategy. DNA fragment encoding the ts mutant *trz1-537 *allele was cloned into an *URA3*-marked integrating vector (p406/*trz1-537*). The resulting plasmid was linearized with Bgl II and transformed into YPH499 to integrate into *TRZ1 *chromosomal locus. Uracil prototrophs were selected from the transformation and plated on FOA to select cells that had undergone excision of the plasmid. The replacement of wild type *TRZ1 *with the ts mutant *trz1-537 *allele was confirmed with sequencing.

YL13-01, YL13-02 and YL13-03, in which the chromosomal *TRZ1 *is deleted and a wild type *TRZ1 *or a *trz1-537 *allele or a *trz1-538 *allele is integrated at the *LEU2 *locus, were generated as follows. DNA fragments encoding *TRZ1*, or the ts mutant alleles *trz1-537 *or *trz1-538 *were cloned into an integrating *LEU2*-marked vector (p405/*TRZ1*). The resultant plasmids were linearized with BstXI, and YL03-47 was transformed with the fragments for integration into the leu2-Δ 1 locus. Leucine prototrophs were selected from the transformation and plated on FOA to select cells that had lost pRS426/*TRZ1*. The identity of the integrated alleles was confirmed by PCR and sequencing.

### Western blot

Yeast cells were grown as indicated in the text. Usually, about 1.5 ml yeast culture with an A_600 _of about 0.6 was centrifuged and the pellet resuspended in 150 μL boiling buffer (50 mM Tris pH8.0, 0.1% Bromophenol blue, 2% SDS, 10% glycerol and fresh 0.1 M DTT). The yeast suspension was heated to 95°C for 20 minutes followed by vortexing and centrifugation at 14000 rpm for 1 minute. The resulting cellular extract was loaded onto a SDS-PAGE gel. Electrophoresis, transfer and Western blot were carried out using Invitrogen's XCell SureLock system following their protocol. Anti-myc and anti-V5 antibodies were purchased from Invitrogen (Calsbad, CA) and used at 1:2000 dilution. Anti-FLAG antibody was purchased from Sigma (St. Louis, MO) and used at 1:400 dilution.

### Temperature-sensitive (ts) mutant screen

To prepare for a screen for *TRZ1 *mutations that rendered yeast growth temperature-sensitive, the codons of specific *TRZ1 *amino acids were mutagenized. We targeted amino acids 535, 536, 537, 538, and 541 for ts screening. To mutagenize amino acid 535, a pair of oligos with degenerate nucleotides encoding residue 535 were synthesized. The primer sequences were as following: 079c.535.F (5'-CAG GAT TTG AAA NNN ATA TAT CTG AGT CAC-3'), 079c.535.R (5'-ACT CAG ATA TAT NNN TTT CAA ATC CTG AAA TAT TG-3'), 079c.NdeI (5'-GAT TAT GAT TGT GCT GAG CTT GGC-3'), 079c.BglII (5'-TTC TTC ACG GCA TCC TCC AGT AGC-3'). Two fragments were generated by primary PCR with wild type *TRZ1 *coding sequence as template and 079c.NdeI/079c.535.R and 079c.535.F/079c.BglII as primers. About 100 ng of each of the resulting fragments were mixed and used as templates for secondary PCR using 079c.NdeI/079c.BglII as primer. The resulting PCR products were purified by Qiagen PCR column, mixed with p415/*TRZ1 *(Δ NdeI-NcoI) that lacks *TRZ1 *sequence between Nde I and NcoI sites, and the mixture used to transform competent YL03-47. Transformants were plated onto SC-LEU. For other targeted amino acids, PCR-generated mini-libraries were made by following a similar strategy to that outlined above. About 500 colonies were screened for each targeted amino acid. After two days' growth on SC-Leu media at 30°C, colonies were replica-plated onto two SC-Leu+FOA plates, one of which was incubated at 30°C, while the other was incubated at 37°C. After 24 hours colonies that grew at 30°C but failed to grow at 37°C were isolated from the original SC-LEU plate. The LEU2 marked plasmids were isolated, sequenced, and used to retransform YL03-47. Transformants were plated on FOA to select yeast that had lost p426/*TRZ1 *and streaked onto SC-LEU+FOA plates to confirm the temperature sensitive phenotype.

### High copy suppressor screen

A 2 μ *URA3*-marked yeast genomic library (gift from Mike Forte and Beth Lachlyd, Vollum Institute, Portland, OR) was used to transform YL12-01. Transformants were plated onto SC-URA and after four days' incubation at 30°C, colonies were picked. About 90 colonies were picked out of a total of 70,000 colonies screened. The library plasmids were isolated and used to retransform YL12-01. The suppressor phenotype was confirmed by comparing the growth of YL12-01 transformed with each library plasmid with that of YL12-01 transformed with p416/Gal1 and p416/*TRZ1*. The sequence of the high copy suppressors was determined.

## List of abbreviations used

*TRZ1*: tRNase Z 1

tRNase Z: transfer RNA (tRNA) 3' processing endoribonuclease

CTD: C-terminal domain

REX: RNA exonuclease

tRNA: transfer RNA

rRNA: ribosomal RNA

snRNA: small nuclear RNA

snoRNA: small nucleolar RNA

## Authors' contributions

YC (Yang Chen) designed most of the experiments, carried out most of the genetic studies, interpreted the data and wrote the manuscript. AB carried out most of the high copy suppressor screens and ts screens. CD carried out most of the growth assays and confirmation of high copy suppressors. YC (Yuan Chen) carried out some of the ts screen and growth assays. DS and SVT have made substantial contributions to conception and design of the experiment and drafting of the manuscript.

## Figures and Tables

**Table 1:** Strains used in this study

YPH499	*MAT*a *ura3-52 lys2-801*^amber ^*ade2*-*101*^orchre ^*trp1-*Δ*63 his3-*Δ*200 leu2-*Δ*1*	ρ^+^
YPH500	Same as YPH499 except the mating type is α	ρ^+^
YPH501	Same as YPH499 except the mating type is a/α	ρ^+^
YL03-47	YPH499 *trz1*Δ*::hisG *(*TRZ1 URA3 *2 μ)	ρ^+^
YL09-00	YPH499 transformed with *Gal1p*-β-*gal (TRP1, CEN)*	NA
YL09-01	YPH499 transformed with *Gal1p-TRZ1 (TRP1, CEN)*	ρ^+^
YL09-02	YPH499 transformed with *Gal1p-FLAG•TRZ1 (TRP1, CEN)*	ρ^+^
YL10-01	YPH500 *trz1*Δ*::URA3 (GAL1p-TRZ1 (TRP1, CEN))*	ρ^-^
YL10-02	YPH499 *trz1*Δ*::hisG (GAL1p-TRZ1 (TRP1, CEN))*	ρ^-^
YL10-03	YPH499 *trz1*Δ*::hisG (GAL1p-FLAG•TRZ1 (TRP1, CEN))*	ρ^+^
YL10-04	YPH499 *trz1::Gal1-FLAG•TRZ1*	ρ^-^
YL10-05	YPH499 *trz1::Gal1p-TRZ1*	ρ^-^
YL12-01	YPH499 *trz1*Δ*::trz1-537*	ρ^-^
YL12-02	YL12-01 transformed with *TRZ1 *(*URA3, CEN)*	ρ^+^
YL13-01	YPH499 *trz1*Δ*::hisG TRZ1 (WT)::LEU2*	ρ^+^
YL13-02	YPH499 *trz1*Δ*::hisG trz1-537::LEU2*	ρ^-^
YL13-03	YPH499 *trz1*Δ*::hsiG trz1-538::LEU2*	ρ^-^

**Table 2:** doubling time (hour)

**Strains**	**SC glucose**	**SC galactose**
**YPH499**	2.18	3.11
**YL09-00**	2.11	2.99
**YL09-01**	2.20	3.85

**YPH499**	2.62	3.75
**YL10-04**	7.70	139
**YL10-05**	5.64	49.5
**YL09-01**	2.22	3.55

**YPH500**	2.27	5.54
**YL10-01**	2.95	63.0

**YPH499**	1.90	3.25
**YL10-01**	2.96	105
**YL10-02**	2.75	431
**YL10-03**	1.83	3.17
**YL03-47**	2.27	3.25

Yeasts were inoculated into 2 mls of SC-raffinose medium and grew overnight at 30°C. The overnight culture was then diluted with either SC-glucose or SC-galactose to O.D. 600 nm of 0.1 and return to 30°C shaking. The O.D. 600 nm reading on these cultures were monitored throughout the day until they reached more than 1. The doubling time equals to ln (2)/k, where k is the slope of the least-squares linear fit of ln (O.D.600 nm) versus time. Spaces between lines in the table separate experiments done on different days.

**Table 3:** growth comparison on different sugar

	**Glucose**			**Sucrose**		
	
	**RT**	**30°C**	**37°C**	**RT**	**30°C**	**37°C**
**YPH499**	+++++	++++	+++++	++++	+++++	+++++
**YL09-01**	+++++	++++	+++++	++++	+++++	+++++
**YL10-01**	+++++	+++	+++	++	++++	++
**YL10-02**	+++++	+++	+++	++	++++	++
**YL10-03**	+++++	++++	+++++	++++	+++++	+++++
**YL03-47**	+++++	++++	+++++	++++	+++++	+++++

	**Raffinose**			**Galactose**		
	
	**RT**	**30°C**	**37°C**	**RT**	**30°C**	**37°C**

**YPH499**	+++	+++++	+++	++	+++++	++
**YL09-01**	+++	+++++	+++	++	+++++	++
**YL10-01**	+	++	+	-	-	-
**YL10-02**	+	++	+	-	-	-
**YL10-03**	+++	+++++	+++	++	+++++	++
**YL03-47**	+++	+++++	+++	++	+++++	++

SC-glucose: 3 days at RT, 2 days at 30 and 37°C.

SC-sucrose: 3 days at RT, 2 days at 30 and 37°C.

SC-raffinose: 3 days at RT, 2 days at 30 and 37°C.

SC-galactose: 4 days at RT, 2 days at 30 and 37°C.

+++++ fully grown spot with pink color

++++ fully grown spot with weak pink color

+++ fully grown spot with white color

++ clearly seen by eye

+ barely seen by eye

Yeasts were inoculated into 2 mls of SC-raffinose medium and grew overnight at 30°C. The resultant overnight cultures were diluted with SC-raffinose medium to O.D. at 600 nm 0.2 and returned to growth at 30°C for another 2–4 hours to get to the exponential growth phase. Then the cultures were diluted to O.D. 600 nm 0.2. These freshly diluted cultures were serially diluted and spotted onto SC plate with different sugars added.

**Table 4:** Complementation of *trz1*

Constructs	Complement Δ trz1
ELAC2	No
ELAC2.FLAG	No
FLAG. ELAC2.C-term	No
ELAC2.N-term	No
ELAC2 (S217L)	No
ELAC2 (A541T)	No
ELAC2 (R781H)	No
ELAC2 (1641insG)	No
ELAC1	No
TRZ1 WT	Yes
FLAG.*TRZ1 *WT	Yes
*trz1 *M535T	Yes
FLAG.*trz1 *M535T	Yes
*trz1 *H542Ter	No
FLAG.*trz1 *H542Ter	No
*trz1 *G548R	No
FLAG.*trz1 *G648R	No
*trz1 *G229D	Yes
*trz1 *Q230A	Yes
*trz1 *G698D	No
*trz1 *D699A	No
FLAG.*TRZ1*. C-term	No
FLAG.*TRZ1*. N-term	No
FLAG.*TRZ1*- ELAC2	No
ELAC2-*TRZ1*	No

The chimeric proteins are made by fusion of either the ELAC2 N-domain with TRZ1 C-domain or the reverse. Trz1p N-domain ends at a.a. 463 and the C-domain starts at a.a. 465. The ELAC2 N-domain ends at a.a. 481 and the C-domain starts at a.a. 481.

The shuffle experiment is done in strain YL03-47 according to protocols described in [32]. The constructs are TRP -marked CEN vectors.

**Table 5:** Doubling time of ts allele integrants

Strain	*LEU2 *locus	RT	37°C
YL13-01	TRZ1 WT	5.54 ± 0.26	1.44 ± 0.04
YL13-02	*trz1-537*	6.87 ± 0.85	2.88 ± 0.01
YL13-03	*trz1-538*	6.31 ± 1.03	4.87 ± 1.15

Strain	*TRZ1 *locus	RT	37°C

YPH499	*WT*	3.17	1.85
YL12-01	*trz1-537*	4.75 ± 0.06	3.67 ± 0.02

Yeasts were inoculated into 2 mls of SC-glucose medium and grew at 30°C overnight. The overnight cultures were diluted with SC-glucose medium to O.D. at 600 nm 0.1 and returned to 30°C with shaking. The O.D. 600 nm reading was monitored throughout the day until it was more 1. Doubling time was calculated as described in table 2.
